# Availability of the Great Saphenous Veins as Conduits for Arterial Bypass Surgery in Patients with Varicose Veins

**DOI:** 10.3390/jcm13247747

**Published:** 2024-12-18

**Authors:** Veronika Golovina, Vladislav Panfilov, Evgenii Seliverstov, Darina Erechkanova, Igor Zolotukhin

**Affiliations:** 1 Department of Fundamental and Applied Research in Vascular Surgery, Pirogov Russian National Research Medical University, 119049 Moscow, Russia; vladipanfilov@gmail.com (V.P.); flebolog@rambler.ru (E.S.); zoloto70@bk.ru (I.Z.); 2 Pirogov City Clinical Hospital No. 1, 119049 Moscow, Russia; darinaere@gmail.com

**Keywords:** varicose veins, vein saving surgery, hemodynamic correction, atherosclerosis, peripheral artery disease, arterial disease

## Abstract

**Background:** The great saphenous vein (GSV) has long been recognized as the best conduit for vascular bypass procedures. Concomitant varicose veins disease may be a reason for GSV unavailability either due to dilatation and tortuosity of the vein or due to its destruction during invasive venous treatment. **Objectives**—to assess the rate of varicose vein patients with concomitant lower extremity arterial disease (LEAD) who have previously lost their GSV due to venous ablation. **Material and Methods:** A total of 285 patients (76 F, 209 M) with LEAD were consecutively enrolled. A total of 111 patients (222 limbs) underwent a detailed duplex ultrasound of the lower extremity veins for assessing suitability of the GSV as a conduit. We registered presence of varicose veins (VVs), type of previous invasive procedure and availability of saphenous veins as possible grafts. **Results:** The mean age of screened patients was 70.5 ± 9.1.62 (21.75%) patients had varicose veins or were operated on before due to varicose veins. A total of 42 patients with varicose veins had C2 disease, 10 had C3, 9 had C4 and 1 had C6 according to CEAP classification. A total of 222 lower extremities were examined by duplex ultrasound of which 51 limbs had VVs. Despite the presence of varicose tributaries, the GSV was suitable for bypass in 9 of those lower extremities. The GSV was not available as a conduit in 34 (19.9%) ipsilateral lower extremities in the LEAD with no VVs group and in 42 (82.6%) ipsilateral lower extremities in the LEAD with VVs group (*p* = 0.0001). Varicose vein disease was associated with a higher frequency of the GSV unavailability (odds ratio 18.8, 95% confidence interval 8.35–42.35). On the 11 ipsilateral limbs (5% of LEAD patients and 21.6% of LEAD with VVs patients), the GSV was unavailable due to previous venous interventions. **Conclusions:** Almost 20% of patients may have both LEAD and VVs. Among those with VVs, most have the ipsilateral GSV unavailable as a potential conduit. Additionally, one fifth of limbs with VVs had GSVs destroyed previously due to saphenous ablative procedures.

## 1. Introduction

Significant progress has been made in the treatment of lower extremity arterial disease (LEAD). An active approach for managing critical limb ischemia includes surgical restoration of the lower extremities blood supply [1]. The development of new generations of vascular prostheses and improvements in bypass and endarterectomy techniques have led to a reduction in mortality, amputation and impaired quality of life. However, the short-term and long-term results of surgical correction of arterial blood flow are mainly determined by the state of the poor distal arterial run-off and the high resistances in the distal arterial network (e.g., the pedal/distal leg arterial network) where the bypass connects and brings flow [2]. The patency of the bypass graft depends on various factors. The most important are the type of operation, the material from which the prosthesis is made, the length of the conduit and the patient’s coagulation conditions [3,4]. It is generally accepted that blood flow contributes the most to conduit failure [1,2].

The results of reconstructive surgery below the inguinal ligament depend on the used material. It is crucially important for below-knee bypasses. Prosthetic grafts have been shown to be inferior to autogenous conduits for infragenicular reconstructions [5,6]. The current guidelines of the European Society for Vascular Surgery outline that availability and quality of an autologous vein conduit are key for successful bypass surgery [2]. The guidelines of the European Society of Cardiology (ESC) on the diagnostics and treatment of peripheral artery disease (PAD) recommend the autologous saphenous vein as the conduit of choice for femoropopliteal bypass (Grade IA). Moreover, it is recommended to prefer the great saphenous vein (GSV) for infra-popliteal arteries bypass (Grade IA) [1]. The latest guideline for the management of lower extremity peripheral artery disease of the American College of Cardiology also confirms this fact [7]. This recommendation is based not only on the fact that the autologous vein shows better patency in the infra-inguinal position than prostheses but also because it is more resistant to postoperative infectious complications [3]. An autologous vein graft from the GSV of the same limb shows the best patency and preservation of the limb compared to other autologous veins [3]. However, there are several patients with critical limb ischemia who need an infra-inguinal bypass but do not have a GSV on the same limb. This is usually due to previous coronary artery bypass grafting (CABG), femoropopliteal proximal or distal bypasses that have already been performed [8]. The vein may have a small caliber or be damaged as a result of superficial thrombophlebitis. Overall, 20–45% of LEAD patients may have no GSV for bypass [9,10,11].

One of the reasons for GSV unavailability is its previous ablation in patients with concomitant varicose veins [6,7,8]. In the last two decades, there has been a significant increase in the number of endovenous ablations of saphenous trunks in patients with varicose veins and a growing number of young and middle-aged individuals who have undergone ablative venous procedures may face problems with venous conduits when they become elderly [12].

Aim—to assess the rate of varicose veins patients with concomitant LEAD who have previously lost their GSV due to venous ablation.

## 2. Material and Methods

### 2.1. Study Design

This was a single-center study conducted on symptomatic patients with LEAD referred to a vascular surgery department for consultation. Patients were consecutively enrolled from December 2023 to May 2024.

### 2.2. Inclusion and Exclusion Criteria

All the patients of both sex having LEAD affecting one or both legs were included. Only those who refused to sign an informed consent were excluded.

### 2.3. Data Collection

Demographic data and medical history were collected. A physical examination was performed. All patients underwent duplex ultrasound examinations of the lower extremity arteries. For the diagnosis of chronic limb-threatening ischemia (CLTI), an established PAD in association with ischemic rest pain or tissue loss was required. Pain must have been present for more than two weeks and associated with an abnormal ankle brachial index (ABI) < 0.4. Patients with CLTI who were scheduled for revascularization also underwent computed tomography angiography of the aorta and lower extremity arteries. The Rutherford classification was used to describe clinical status. Patients with varicose veins were described according to the CEAP classification. Personal histories of deep vein thrombosis and superficial thrombophlebitis and previous venous invasive treatments were collected.

All patients who met the inclusion criteria were presented to our clinic with data obtained from duplex ultrasound examinations of the arteries and veins of the lower extremities conducted in primary care settings. Generally, such examinations confirm the presence of arterial or venous pathology but do not provide important details regarding the condition of superficial veins. The data obtained from this cohort of patients were used to assess the magnitude of the problem, specifically the coincidence of LEAD and varicose veins, as well as to describe demographic characteristics and general disease profiles.

To evaluate the suitability of the GSV, additional duplex ultrasound examinations were performed on 111 patients. A thorough examination of the lower extremity veins, essential for a clear analysis of the presence and suitability of the GSV as a conduit, was conducted by a vascular specialist experienced in venous assessments. This sub-cohort was utilized to evaluate the presence and suitability of the GSV as bypass material.

Duplex ultrasound was performed to assess the presence of varicose veins, consequences of previous invasive procedures and availability of saphenous trunks as potential grafts. Duplex ultrasound was performed in a standing position to access deep veins and GSV. We evaluated the competence of the GSV terminal valve, the presence of reflux in the GSV (≥0.5 s) and deep vein reflux (≥1 s). All measurements of the diameter of the GSV were conducted with transversal position of ultrasound probe.

The primary outcome measure was to determine the number of ipsilateral limbs with absent GSV due to previous interventions in patients with varicose veins and LEAD. Secondary outcome measures included determining the number of ipsilateral limbs without usable GSV due to varicosity, as well as the total number of ipsilateral limbs with absent GSV for all causes (including post-thrombotic changes, stripping, previous stripping or thermal/non-thermal ablation or small vein diameter).

We found no criteria defining the suitability of a vein as a bypass graft in previously published papers. We considered GSV as available if the vein diameter was 4 or more mm and no varicosity transformation and/or post-thrombotic changes due to superficial thrombophlebitis were registered. We considered the vein with reflux and enlarged diameter suitable for bypass with varicose changes limited to tributaries. The absence of GSV due to GSV stripping, endovenous thermal or non-thermal ablation, as well as previous CABG or femoropopliteal bypass, was registered. Considering that the GSV is the conduit of choice for bypass, the study focused on the GSV only.

### 2.4. Approval

The study protocol was approved by the Ethical Committee of Pirogov Russian National Research Medical University (No. 235, 18 December 2023) and registered on clinicaltrials.gov as NCT06332833.

### 2.5. Data Analysis

Statistical analysis was performed using SPSS 23 software. Descriptive data are presented as *n* (%) for categorical variables and as mean ± SD. Continuous variables were tested for normality using the Kolmogorov–Smirnov test and presented as mean ± SD. Student’s *t* tests and Mann–Whitney U test were used to test continuously distributed variables. Categoric variables were compared by Pearson’s chi-squared test or Fisher exact test. Values of *p* < 0.05 were considered significant for all tests.

## 3. Results

A total of 285 patients with LEAD were assessed for eligibility. Of these, 209 (73.3%) were male and 76 (26.7%) were female, with a mean age of 70.5 ± 9.1 years. There were no patients with previous deep vein thrombosis ot superficial vein-thrombosis. Sixteen patients had a history of superficial vein thrombosis. Distribution of LEAD severity according to Rutherford classification is shown in Table 1.

Among all the patients assessed, 62 (21.75%), including 44 males and 18 females, had varicose veins (13%) or had undergone previous varicose vein-related procedures (8,75%). The mean age of this cohort was 73.8 ± 10.5. A total of 42 patients had C2 disease, 10 had C3, 9 had C4 and 1 had C6 according to CEAP classification.

Detailed duplex ultrasound of the veins of the lower extremities was performed on 111 patients (222 lower extremities). Of these, 83 (74.8%) were male and 28 (25.3%) were female, with a mean age of 69.9 ± 8.7 years. Of them, there were 32 patients with VVs presented on 51 limbs. The mean age of this cohort was 72.4 ± 8.4. 41 limbs were classified as C2, 5 as C3, 4 as C4 and 1 as C6 according to CEAP classification. Some characteristics of patients with and without VVs who had undergone detailed duplex ultrasound are presented in Table 2.

The total number of lower limbs lacking an ipsilateral GSV was 76 (34.2%). The absence of GSV as a conduit was significantly higher in the LEAD with varicose veins group (Table 3).

Varicose vein disease was associated with a higher frequency of the GSV unavailability (odds ratio 18.8, 95% confidence interval 8.35–42.35). Despite the presence of varicose tributaries, the GSV trunk was suitable for a bypass in nine of the lower extremities.

## 4. Discussion

We conducted a single-center cross-sectional study on consecutive symptomatic LEAD patients. The primary objective of our study was to evaluate the impact of venous interventions in patients with varicose veins on the availability of the GSV as a potential conduit for future bypass surgery.

Almost a quarter of individuals with LEAD have concomitant varicose veins disease. We found that the ipsilateral GSV was overall unavailable in a third of LEAD patients. Presence of VVs disease leads to frequent ipsilateral GSV unavailability. On limbs with VVs, an ipsilateral GSV was unavailable in 82.6% cases compared with 20% of limbs without VVs.

The most common GSV was dilated and/or tortuous, which was observed in a half of lower extremities with VVs. In 21.6% of LEAD with VVs patients and 5% of LEAD both with and without VVs patients, the GSV was unavailable due to previous venous interventions. Two patients lost their GSVs after venous interventions on both legs. One of them was a 65-year-old male with LEAD, who had occlusion of the superficial femoral artery in both legs, classified as Rutherford stage 3. The patient had previous endovenous laser ablation of both GSVs 10 years ago. Additionally, he underwent a coronary artery bypass grafting (CABG) procedure using the radial artery 5 years ago due to the lack of suitable venous conduits. In this clinical scenario, the absence of a venous conduit for bypass surgery in the lower extremities poses a significant threat of limb loss.

Among the cohort of patients with LEAD and concomitant varicose veins disease, only three patients presented with critical limb ischemia, necessitating revascularization procedures. Two of these patients were then operated on by percutaneous transluminal angioplasty of the superficial femoral artery, while one patient received a bypass grafting procedure using the contralateral GSV. Most patients in our study did not require bypass procedures at the time of inclusion. However, they may become candidates for such interventions in the near future. Nonetheless, some of these patients may lose their GSV due to the endovenous treatment of varicose disease during this period if they visit a venous clinic focused on performing thermal ablations.

We found only two papers with similar data presented. Taylor LM et al. reported GSV unavailability in 43% of PAD patients. The GSV was absent because of previous vein stripping or previous utilization as a bypass conduit in 23% limbs and in 20% due to small size (diameter less than 4 mm) [10]. Chew D.K.W. et al. found no GSV suitable for bypass in about 20% of the cases, with no exact information of what were the reasons for vein unavailability [9]. A similar rate was found in our study.

The contralateral GSV can be utilized as a bypass conduit; however, this approach may compromise the availability of venous material in the opposite limb, which could be necessary for future surgical interventions. In cases where the GSV is absent, alternative sources such as the small saphenous vein (SSV), arm veins or distal segments of the GSV in the leg—after thermal ablation limited to the thigh—may still serve as components in a composite graft. Nevertheless, autologous vein grafts harvested from the GSV of the same limb demonstrate superior patency rates and better preservation of limb function compared to other autologous venous sources [13]. Consequently, the preservation of the GSV is a critical consideration in surgical planning.

With the growing life expectancy, the number of patients in need of bypass surgery will increase. Almost a quarter of the population in developed countries are older than 65 years: 36% in Monaco, 30% in Japan, 24% in Italy, 23% in Finland, 22 in Germany, etc. [14]. Due to that, the number of patients with LEAD increases. Annually, the total number of interventions due to LEAD in USA only increased by 15% between 2001 and 2007 (106,018 vs. 121,596, *p* < 0.001) [15,16].

On the other hand, in the last two decades, the number of venous interventions performed on VVs patients has severely increased. The first to pay attention to this was P. Lawrence [17]. His report showed that from 1996 to 2014, the number of venous ablation procedures in the USA increased by 4529% [18]. This information was so horrifying that Russell Samson, editor of Vascular Specialist, in irony proposed a new society titled “Save Our Saphenous” [19].

Crawford J.M. et al. extracted data on practice trends in endovenous ablation from the Medicare Data Utilization and Payment Database [12]. It has been shown that there is a steady increase in the number of patients undergoing ablation from 2012 to 2015. The number of procedures grew from 132,200 to 170,033, and the number of patients from 74,333 to 91,441. Procedures per patient averaged 1.8 in the aggregate dataset. The total number of providers performing more than two ablations per patient on average almost doubled from 301 to 511. Moreover, the number of ablations per patient was higher if the ablation was performed by a physician with no vascular training. P. Lawrence suggested that this situation could be due to the fact that some physicians may not be aware of the published literature and practice guidelines, or may not be proficient in duplex ultrasound imaging and reflux valuation [20].

According to the Cochrane database of systematic reviews on interventions for GSV incompetence, the mean age of people who underwent venous intervention was about 50 years [21]. In our research, the mean age of patients who had come to our clinic with LEAD was 70.5 ± 9.1. This means that people who underwent venous intervention in 2012–2016 are not yet at the age of LEAD manifestation and they may face problems with venous conduits in the next decade when they become elderly. Furthermore, the problem with the GSV destruction may increase rapidly in the opinion of some specialists that patients who have symptomatic C1 patients with refluxing saphenous veins without visible varicose veins could be candidates for an ablation procedure [22]. Another issue is related to the cases when thermal ablation is recommended even to C1 patients whose saphenous veins are fully intact. While these malpractice examples are often seen by many venous specialists, not that many literature sources discuss the problem. Marc A. Passman, former president of the American Venous Forum, presents an excellent example of a young woman who was recommended to have thermal ablation on six saphenous veins on both legs while having all of them competent and complaining only about spider veins presence [23].

There are four fundamental principles that must be upheld for medical practice to be considered ethical. These principles include autonomy, which ensures that patients have the freedom of thought, intention and action, allowing them to make healthcare decisions without coercion; justice, which emphasizes the equitable distribution of both the burdens and benefits of new treatments across all societal groups; beneficence, which mandates that medical procedures are performed with the intention of promoting the patient’s well-being; and nonmaleficence, which requires that procedures do not inflict harm on the patient or others in society [24]. Regrettably, current practices in varicose vein management are influenced by several non-medical factors which can have an effect on the ethical status of a physician. Ricci, Mendoza and De Maeseneer, respectively, remark with emphasis that “new gadgets will be continuously invented, leaving unchanged the GSV closure rate”, and “in lots of countries, the health professionals’ income depends on their performance. The higher the income for a procedure, the higher the personal financial benefit the more the cost, the better the treatment, the better the income” [25,26].

Summing up, vascular surgeons will see much more patients with a destructed GSV in the coming years. That is why it may be worth taking a closer look at hemodynamic approaches such as CHIVA and the ambulatory selective variceal ablation under a local anesthesia (ASVAL) technique. Claude Franceschi was the first to suggest the possible need to preserve the main trunks of the saphenous veins and proposed the ambulatory conservative hemodynamic correction of venous insufficiency (CHIVA) method [27]. Subsequently, followers of his technique P. Zamboni, S. Gianesini and E. Mendoza supported this method and the possibility of using the preserved saphenous veins as future bypass [28]. A hemodynamic approach means eliminating the escape point, saving re-entry points and preserving saphenous trunks [29]. By maintaining the integrity of the great and/or small saphenous veins, this approach ensures continued drainage of superficial tissues. Moreover, there are other advantages of this method such as allowing local anesthesia, not requiring hospitalization, immediate ambulation, lowering costs, being easily repeatable and being ethically correct. A systematic review conducted by Cochrane has demonstrated that CHIVA is as effective as traditional stripping methods and may also lead to lower rates of nerve injury and bruising [30]. Most studies show that this technique could not only help to preserve the trunk of the saphenous veins but also decrease the diameter of the GSV to normal calibers [31,32,33,34].

In its traditional implementation, CHIVA involves the open ligation of the great saphenous vein (GSV) at the saphenofemoral junction (SFJ) [27]. However, this approach has become less favorable with the advent of endovenous techniques. To address the limitations associated with open surgical methods, S. Gianesini and colleagues proposed a combined approach that integrates endovenous ablation with the principles of CHIVA [35]. The procedure focuses on ablating only a specific segment of the GSV trunk near the SFJ, thereby preserving the trunk while effectively eliminating reflux from the deep venous system. This innovative combination was initially referred to as the hot-CHIVA.

On the other hand, hemodynamic correction does not mean that GSV is saved for a long time period. In the first year after hot-CHIVA, the recurrence of varicose veins was 4.9%, which is similar to standard RFA [34]. CHIVA in two steps or “CHIVA 2” with ligature flush between the GSV and varicose tributaries as a possible alternative intervention sparing GSV trunk, also known as an ASVAL procedure, showed less encouraging results. The recurrence after an ASVAL procedure after 1 year was 10.8%, after 2 years 22.7%, after 3 years 33.1%, after 4 years 46.5%, and after 5 years 66.3% [21,36]. Even if a hemodynamic approach leads to the same results in the long run, it will have the advantage of saving the GSVs as conduits for future needs. The fact that 21% of LEAD patients’ limbs with VVs had GSVs destructed before makes the idea of CHIVA proponents at least worth discussing.

Considering all these aspects, the treatment of varicose veins disease, particularly in patients with LEAD, should be approached with special significance and responsibility by the operating surgeon. While it is a subject of debate, in this patient population, it may be possible not only to preserve veins through hemodynamic correction methods but also to limit GSV ablations in refluxing veins with minimal dilation; avoiding the elimination of trunks measuring 4 mm, 5 mm and even 6 mm, when uncomplicated, could be a viable consideration.

### Limitations

There are several limitations to our study. These include the relatively small sample size and the inability to confirm the availability of the GSV in all patients due to the lack of duplex ultrasound during consultations. In our study, we considered the GSV suitable for bypass grafting if its diameter was 4 mm or greater. However, there is evidence suggesting that smaller diameter superficial veins can also be used as conduits, albeit with less favorable prognostic outcomes. Additionally, this research was conducted at a single hospital only. We also included only patients who actively seek medical help. Approaches to VVs ablation are also different in other countries so they may have another magnitude of problem.

## 5. Conclusions

Both LEAD and VVs diseases can be found in about one fifth of patients. Among those who have VVs, the majority have an ipsilateral GSV unavailable as a possible conduit. A total of 20% of limbs with VVs veins had GSVs totally destroyed before due to saphenous ablative procedures.

## Figures and Tables

**Table 1:** Stage of LEAD according to Rutherford classification.

Category	All Patients, *n* = 285	Male, *n* = 209	Female, *n* = 76
1	66 (23.2%)	47 (22.5%)	19 (25%)
2	84 (29.5%)	64 (30.6%)	20 (26.3%)
3	105 (36.8%)	78 (37.3%)	27 (35.5%)
4	16 (5.5%)	11 (5.3%)	5 (6.6%)
5	14 (5%)	9 (4.3%)	5 (6.6%)

**Table 2:** Demographics and clinical characteristics of both groups’ patients.

	Group		*p*
	LEAD Only (*n* = 79)	LEAD with VV (*n* = 32)	
Age	69 ± 10.9	72.3 ± 10.1	0.825
Female	16 (20%)	12 (37.5%)	0.058
Male	63 (80%)	20 (62.5%)	
Diabetes	22 (27.8%)	9 (28.1%)	0.976
Smoking	58 (73.4%)	18 (56.3%)	0.078
Diabetes plus smoking	10 (12.6%)	3 (9.3%)	0.062

**Table 3:** Unavailability of GSV on the ipsilateral lower extremity (limbs, *n* = 222).

	Group		*p*
	LEAD (*n* = 171)	LEAD with VVs (*n* = 51)	
GSV unavailable	34 (19.9%)	42 (82.6%)	0.0001
Small size (less than 4 mm)	24 (14%)	1 (2%)	0.016
Removed for bypass before	10 (5.9%)	-	-
Dilation and/or varicosity	-	24 (47%)	-
Previous ablation of GSV	-	11 (21.6%)	-
Postthrombotic intraluminal changes	-	6 (11.8%)	-

## Data Availability

The raw data supporting the conclusions of this article will be made available by the authors on request.
